# The Anti-fibrotic Actions of Relaxin Are Mediated Through a NO-sGC-cGMP-Dependent Pathway in Renal Myofibroblasts *In Vitro* and Enhanced by the NO Donor, Diethylamine NONOate

**DOI:** 10.3389/fphar.2016.00091

**Published:** 2016-03-31

**Authors:** Chao Wang, Barbara K. Kemp-Harper, Martina Kocan, Sheng Yu Ang, Tim D. Hewitson, Chrishan S. Samuel

**Affiliations:** ^1^Cardiovascular Disease Program, Biomedicine Discovery Institute, Department of Pharmacology, Monash UniversityClayton, VIC, Australia; ^2^Drug Discovery Biology, Monash Institute of Pharmaceutical Sciences, Monash University, ParkvilleVIC, Australia; ^3^Department of Nephrology, Royal Melbourne Hospital, ParkvilleVIC, Australia; ^4^Department of Medicine, Royal Melbourne Hospital, University of MelbourneParkville, VIC, Australia

**Keywords:** fibrosis, myofibroblasts, cell signaling, relaxin, transforming growth factor-β1, nitric oxide, cGMP

## Abstract

**Introduction:** The anti-fibrotic hormone, relaxin, has been inferred to disrupt transforming growth factor (TGF)-β1/Smad2 phosphorylation (pSmad2) signal transduction and promote collagen-degrading gelatinase activity via a nitric oxide (NO)-dependent pathway. Here, we determined the extent to which NO, soluble guanylate cyclase (sGC) and cyclic guanosine monophosphate (cGMP) were directly involved in the anti-fibrotic actions of relaxin using a selective NO scavenger and sGC inhibitor, and comparing and combining relaxin’s effects with that of an NO donor.

**Methods and Results:** Primary renal cortical myofibroblasts isolated from injured rat kidneys were treated with human recombinant relaxin (RLX; 16.8 nM), the NO donor, diethylamine NONOate (DEA/NO; 0.5–5 μM) or the combined effects of RLX (16.8 nM) and DEA/NO (5 μM) over 72 h. The effects of RLX (16.8 nM) and DEA/NO (5 μM) were also evaluated in the presence of the NO scavenger, hydroxocobalamin (HXC; 100 μM) or sGC inhibitor, ODQ (5 μM) over 72 h. Furthermore, the effects of RLX (30 nM), DEA/NO (5 μM) and RLX (30 nM) + DEA/NO (5 μM) on cGMP levels were directly measured, in the presence or absence of ODQ (5 μM). Changes in matrix metalloproteinase (MMP)-2, MMP-9 (cell media), pSmad2 and α-smooth muscle actin (α-SMA; a measure myofibroblast differentiation) (cell layer) were assessed by gelatin zymography and Western blotting, respectively. At the highest concentration tested, both RLX and DEA/NO promoted MMP-2 and MMP-9 levels by 25–33%, while inhibiting pSmad2 and α-SMA expression by up to 50% (all *p* < 0.05 vs. untreated and vehicle-treated cells). However, 5μM of DEA/NO was required to produce the effects seen with 16.8 nM of RLX over 72 h. The anti-fibrotic effects of RLX or DEA/NO alone were completely abrogated by HXC and ODQ (both *p* < 0.01 vs. RLX alone or DEA/NO alone), but were significantly enhanced when added in combination (all *p* < 0.05 vs. RLX alone). Additionally, the direct cGMP-promoting effects of RLX, DEA/NO and RLX+DEA/NO (which all increased cGMP levels by 12-16-fold over basal levels; all *p* < 0.01 vs. vehicle-treated cells) were significantly inhibited by pre-treatment of ODQ (all *p* < 0.05 vs. the respective treatments alone).

**Conclusion:** These findings confirmed that RLX mediates its TGF-β1-inhibitory and gelatinase-promoting effects via a NO-sGC-cGMP-dependent pathway, which was additively augmented by co-administration of DEA/NO.

## Introduction

Fibrosis (organ scarring) is a universal manifestation of chronic or severe tissue injury and represents an aberrant wound healing response, which in effect results from the failure of the affected organ to repair and regenerate (Wynn and Ramalingam, 2012). The pathogenesis of fibrosis in several organs including the kidney involves consecutive but overlapping events in response to injury or stress, including inflammation, extracellular matrix (ECM) synthesis (fibrogenesis) and remodeling (Eddy, 2000; Hewitson, 2009; Wynn and Ramalingam, 2012). The rate of ECM synthesis which contributes to fibrosis is dependent on the activity of various pro-fibrotic cytokines and growth factors, primarily transforming growth factor (TGF)-β1, and on myofibroblast burden (Eddy, 2000; Hewitson, 2009; Wynn and Ramalingam, 2012). Recognized from their *de novo* expression of α-smooth muscle actin (α-SMA), myofibroblasts are mainly derived from the proliferation and differentiation of local fibroblasts following injury, and synthesize excessive amounts of collagen and other ECM proteins that form the basis of renal fibrosis.

In addition to synthesizing ECM components, myofibroblasts also secrete various ECM degrading proteinases, with the extent of ECM accumulation depending upon the balance between ECM synthesis and degradation. The latter can be regulated by several proteases, of which the matrix metalloproteinase (MMP) family, that are in turn regulated by tissue inhibitors of metalloproteinases (TIMPs), are the most studied (Catania et al., 2007; Hewitson, 2009). The gelatinases, MMP-2 (gelatinase A) and MMP-9 (gelatinase B) preferentially cleave basement membrane collagen IV within the kidney, while MMP-2 can also digest interstitial collagens (Aimes and Quigley, 1995). The effect of TGF-β1 on gelatinase activity has been demonstrated to be inhibitory (Howard et al., 2012), but can vary in a time- (Ogawa et al., 2011) and species/model (Saed et al., 2000; Ogawa et al., 2011; Ye et al., 2011; Bates et al., 2015)-dependent manner.

The naturally occurring hormone, relaxin, has emerged as an effective anti-fibrotic in several organs (reviewed in Samuel and Hewitson, 2006; Bennett, 2009; Du et al., 2014) including the aged (Samuel et al., 2004b; Danielson et al., 2006) and injured (Garber et al., 2001, 2003; McDonald et al., 2003; Lekgabe et al., 2005; Hewitson et al., 2010; Sasser et al., 2011; Yoshida et al., 2012, 2013) kidney. Relaxin has been well-demonstrated to inhibit TGF-β1-induced myofibroblast differentiation and myofibroblast-induced aberrant ECM/collagen production (Unemori and Amento, 1990; Unemori et al., 1996; Samuel et al., 2004a; Heeg et al., 2005; Mookerjee et al., 2009), while also being able to augment ECM/collagen degradation through its ability to up-regulate various collagenases (Unemori et al., 1996; Bennett et al., 2003; Kapila et al., 2009; Samuel et al., 2011; Ahmad et al., 2012; Chow et al., 2012; Bennett et al., 2014) and gelatinases (Lekgabe et al., 2005; Kapila et al., 2009; Conrad and Shroff, 2011; Ahmad et al., 2012; Chow et al., 2012; Sassoli et al., 2013; Frati et al., 2015; Sarwar et al., 2015) and/or inhibit TIMP activity (Unemori and Amento, 1990; Williams et al., 2001; Samuel et al., 2008; Sassoli et al., 2013; Bennett et al., 2014).

More recent studies aimed at elucidating the signal transduction mechanisms by which its TGF-β1-inhibitory effects are mediated in (myo)fibroblasts have shown that relaxin signals through its cognate G protein-coupled receptor, relaxin family peptide receptor 1 (RXFP1), to activate an extracellular signal-regulated kinase 1/2 phosphorylation (pERK1/2) and neuronal nitric oxide (NO) synthase (nNOS)-sGC/cyclic guanosine monophosphate (cGMP)-dependent pathway (Mookerjee et al., 2009; Chow et al., 2012; Sarwar et al., 2015). This primarily results in the relaxin-mediated down-regulation of Smad2 phosphorylation (pSmad2) in several organs (Heeg et al., 2005; Mookerjee et al., 2009; Bennett et al., 2014; Royce et al., 2014; Samuel et al., 2014), but also notch-1-induced down-regulation of pSmad3 in cardiac fibroblasts (Sassoli et al., 2013). The inhibition of these intracellular proteins, that promote TGF-β1 signal transduction, leads to suppression of TGF-β1-induced myofibroblast differentiation and aberrant ECM/collagen deposition. Furthermore, by down-regulating TGF-β1 activity at the pSmad2 level, relaxin has been shown to up-regulate iNOS contributing to its MMP-promoting effects (Chow et al., 2012).

As many of relaxin’s actions are mediated via NO (Baccari and Bani, 2008), studies investigating its anti-fibrotic actions have assumed a role for NO given relaxin’s ability to stimulate nNOS in cardiac and renal fibroblasts (Mookerjee et al., 2009; Chow et al., 2012; Sarwar et al., 2015) and/or up-regulate iNOS in renal and dermal fibroblasts (Chow et al., 2012). This is supported by reports of an inverse relationship between NO/cGMP and TGF-β1 (Chu and Prasad, 1999) as well as pSmad2/pSmad3 (Chen et al., 2011) activity. Moreover, both NO (Saura et al., 2005) and direct sGC stimulators (Beyer et al., 2015) have been demonstrated to inhibit TGF-β1 signal transduction. However, the actual involvement of NO in the anti-fibrotic actions of relaxin was not directly demonstrated in those studies. To address this limitation, we investigated the extent to which NO, sGC and cGMP were directly involved in the anti-fibrotic actions of relaxin, by evaluating the effects of a selective NO scavenger (hydroxocobalamin; HXC) and sGC inhibitor (ODQ) on four well-known end-points associated with the anti-fibrotic effects of relaxin: pSmad2, α-SMA (as a measure of myofibroblast differentiation), MMP-2 and MMP-9. Furthermore, we compared and combined the effects of relaxin with a NO donor (diethylamine NONOate; DEA/NO) on these four end-points, and additionally on cGMP levels, in the absence or presence of ODQ.

## Materials and Methods

### Cell Culture

Primary renal cortical myofibroblasts were isolated from fibrotic kidneys of Sprague-Dawley rats 3-days post-ureteric obstruction, characterized and identified as detailed before (Grimwood and Masterson, 2009; Mookerjee et al., 2009; Chow et al., 2012), and used between passages 30–35 (P30–35) for the outlined studies. Cells were re-characterized at the completion of the experiments. Based on cytochemical staining for alpha-smooth muscle actin, 100% of cells were myofibroblasts. Pericytes and smooth muscle cells were excluded by the absence of positive staining for the cytoskeletal protein desmin. Isolating cells from UUO-injured rats was approved by the appropriate Animal Ethics Committee within the University of Melbourne; which adheres to the Australian Code of Practice for the care and use of animals for scientific purposes. It has previously been shown that cells isolated from fibrotic kidneys are more active and a better reflection of their *in vivo* counterparts than fibroblasts that are cultured from normal kidneys (Rodemann and Muller, 1990). These cells are responsive to recombinant human relaxin (RLX) treatment as they specifically express RXFP1 (Masterson et al., 2004; Mookerjee et al., 2009; Chow et al., 2012). Rat renal myofibroblasts were cultured in Dulbecco’s Modified Eagles Medium (DMEM; GIBCO-Life Technologies, Grand Island, NY, USA) containing 10% (v/v) fetal calf serum (FCS; GIBCO-Life Technologies), penicillin (100 units/mL), streptomycin (100 μg/mL), 2% (v/v) 1M HEPES (GIBCO-Life Technologies) and 1% (v/v) 200 μM L-glutamine (Sigma–Aldrich, St Louis, MO, USA) (DMEM-FCS) and were maintained in a 37°C incubator with 95% O_2_/5% CO_2_. The FCS acted as a protein carrier to ensure the stability of rhRLX in the culture media, while the renal fibroblasts were found to grow/proliferate better in the presence of HEPES and L-glutamine. Cells were cultured to approximately 80–90% confluence before being split and passaged or seeded for experiments. All experiments described below were performed at least 3–4 separate times in duplicate.

### Determining the Effects of an NO Donor, NO Scavenger and sGC Inhibitor on the Anti-fibrotic Effects of RLX

To evaluate the direct contributions of NO and sGC to the anti-fibrotic effects of RLX, renal myofibroblasts were seeded in 12-well plates at a density of 1–1.25 × 10^5^ cells/well and treated with either RLX alone (16.8 nM or 100 ng/ml; which is an optimal concentration that had previously been shown to inhibit TGF-β1-induced pSmad2 and α-SMA expression (Masterson et al., 2004; Mookerjee et al., 2009), while promoting MMP-2 and MMP-9 levels (Chow et al., 2012) in these cells, increasing concentrations of the NO donor, DEA/NO (0.1, 1, 5 μM; Sigma–Aldrich) or the combined effects of RLX (16.8 nM) and DEA/NO (5 μM) for 72 h. DEA/NO was initially made up as a 10 μM stock solution in 0.01M NaOH (to prevent DEA/NO from decomposing) and diluted to the appropriate concentrations in DMEM-FCS immediately prior to addition. DEA/NO was added three times daily (at ~5–6 h intervals) over the 72 h treatment-period, while RLX was added once at the beginning of each experiment. Untreated cells and cells treated with 10 μM NaOH (vehicle) alone were used as controls.

In separate experiments, renal myofibroblasts were seeded as above and treated with DEA/NO (5 μM) or RLX (16.8 nM), in the absence or presence of the NO scavenger, HXC (100 μM; Sigma–Aldrich; Favaloro and Kemp-Harper, 2007; Andrews et al., 2009) or sGC inhibitor, ODQ (5 μM; Cayman Chemicals, Ann Arbor, MI, USA; Mookerjee et al., 2009; Chow et al., 2012) for 72 h. HXC or ODQ were added once daily over the 72 h treatment-period, and their effects alone were also evaluated to ensure that they did not affect basal expression of the various end-points measured. After 72 h in each case, the media and cell layers were collected and assayed as described below.

In another set of experiments, renal myofibroblasts were seeded in 48-well plates at a density of 1 × 10^5^ cells/well and grown overnight in DMEM-FCS. Cells were serum starved for 6 h before addition of ligands, then pre-treated with or without ODQ (5 μM) for 15 min prior to stimulation with either RLX [30 nM; a concentration that had previously been used to stimulate cGMP levels in fibroblasts (Sarwar et al., 2015)] alone, DEA/NO (5 μM) alone or the combined effects of RLX (30 nM) and DEA/NO (5 μM) for 30 min. Myofibroblasts treated with vehicle (10 μM NaOH) were used to measure basal cGMP levels. cGMP accumulation was detected using the AlphaLISA cGMP Detection kit (Perkin Elmer, Hopkington, MA, USA). Cells were lysed in ice–cold 100% ethanol following addition of 80 μL of lysis buffer. Twenty microliter lysate was transferred to the 384-well optiplate (PerkinElmer) and analyzed according to the manufacturer’s instructions.

### Protein Isolation and Western Blotting

After the media was removed, the cells within each well were washed with Dulbecco’s Phosphate-Buffered Saline (DPBS) (GIBCO-Life Technologies) to remove any residual media. After discarding the DPBS, cells were then treated with Accutase solution (Sigma–Aldrich, St Louis, MO, USA) and incubated at 37°C for 5 min to facilitate the harvest of cells from the 12-well plates. Harvested cells were then transferred to Eppendorf tubes and centrifuged at 7000 rpm for 10 min. Cell pellets from each well were then re-suspended in 20 μL of RIPA lysis buffer and incubated on ice for at least 30 min for cell lysis. After incubation, the cell lysates were centrifuged at 13200 rpm for 10 min at 4°C, thereafter the supernatants were collected and transferred to new Eppendorf tubes and stored at -80°C until required.

Equivalent one-third portions of each protein sample (for the analysis of pSmad2, total Smad2/3 and α-SMA) were separated on pre-cast 10% SDS-polyacrylamide separating gels (Bio-Rad, Philadelphia, PA, USA) as described before (Chow et al., 2012), and transferred to PVDF membranes using the Bio-Rad Trans-Blot Turbo transfer system (Bio-Rad, Philadelphia, PA, USA), before membranes were blocked for 1 h with 5% (w/v) skim milk powder. Membranes were then probed with primary rabbit monoclonal antibodies to either total Smad2/3 (#3102; 1:1000 dilution; Cell signaling, Danvers, MA, USA) or pSmad2 (#3108; 1:1000 dilution; Cell signaling), or with a primary mouse monoclonal antibody to α-SMA (M0851; 1:1000 dilution; DAKO, Glostrup, Denmark) for at least 16 h at 4°C. Membranes were then probed with a goat anti-rabbit antibody (1:2500 dilution; DAKO) to detect total Smad2/3 and pSmad2; while a goat anti-mouse antibody (1:2000 dilution; DAKO) was used to detect α-SMA. Membranes were then developed with the Bio-Rad Clarity Western ECL substrate kit (Bio-Rad) for 5 min according to the manufacturer’s protocol followed by visualization and imaging using the Bio-rad ChemicDoc MP. The optical density (OD) of the appropriate bands were then quantified and analyzed using Image Lab software (Bio-Rad). The measured density of pSmad2 and α-SMA were then corrected to the density of total Smad2/3, expressed as the relative ratio to that measured from the untreated or vehicle-treated control group (which was expressed as 1 in each case), and subsequently presented as the mean OD for pSmad2 or α-SMA from the *n* = 3–4 separate experiments performed per treatment group.

### Gelatin Zymography

Changes in latent (L) and active (A) MMP-2 and MMP-9 levels that were secreted from rat renal myofibroblasts into the cell media over the 72 h experimental period, were assessed by gelatin zymography as described previously (Chow et al., 2012). Equal volumes of each sample were electrophoresed on 7.5% acrylamide gels containing 1mg/ml gelatin. Gelatinolytic activity was indicated by clear bands and densitometry of these MMP bands was performed using the Bio-rad ChemicDoc MP and Image Lab software (Bio-Rad). The mean ± SEM of each (combined latent and active) MMP was then determined and expressed as the relative ratio to that measured from the untreated or vehicle-treated control group (which was expressed as 1 in each case); from the *n* = 3–4 separate experiments performed per treatment group.

### Statistical Analysis

All statistical analyses was performed using GraphPad Prism v6.0 (GraphPad Software Inc., La Jolla, CA, USA). All data were expressed as the mean ± SEM and analyzed by one-way ANOVA, using a Neuman–Keuls *post hoc* test to allow for multiple comparisons between groups; while differences in cGMP levels were analyzed by Student’s *t*-tests. *P* < 0.05 was considered statistically significant.

## Results

### The Individual vs. Combined Effects of RLX and DEA/NO on Measures of Renal Fibrosis

**FIGURE 1:**
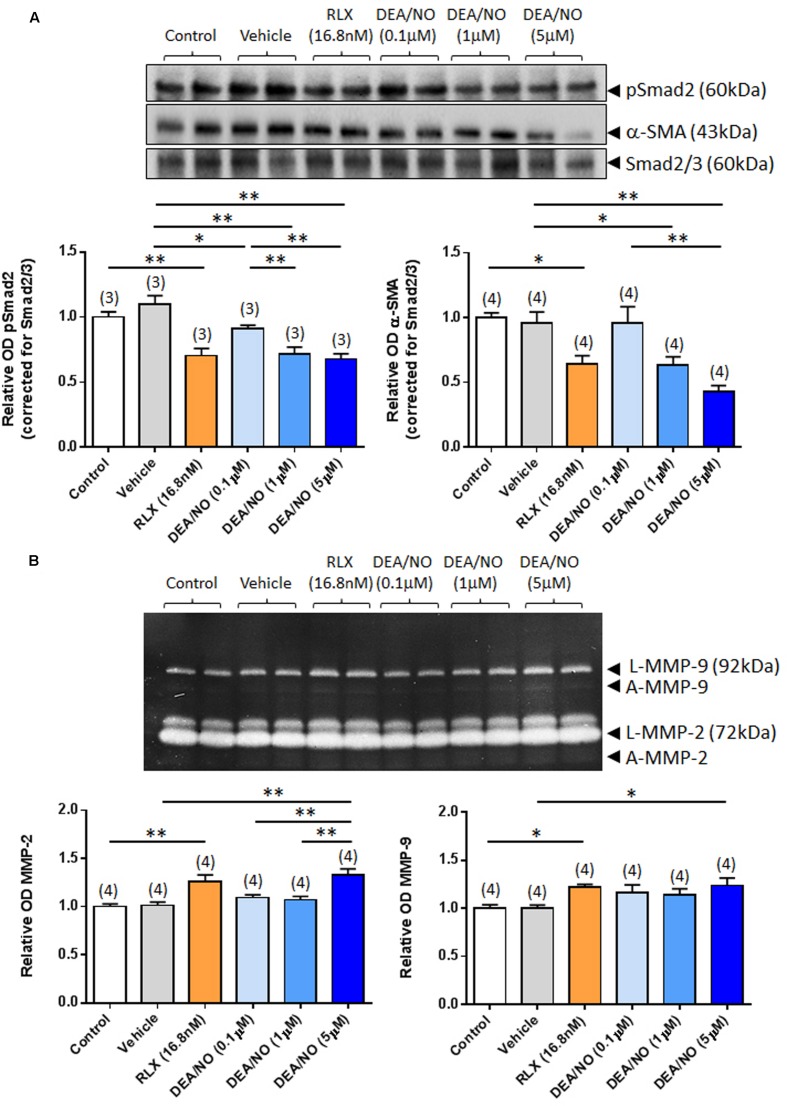
**The effects of RLX vs. DEA/NO on pSmad2, α-SMA, MMP-2 and MMP-9.** Shown are **(A)** representative (duplicate samples from one experiment) Western blots of pSmad2 and α-SMA; and **(B)** gelatin zymographs of latent (L) and active (A) MMP-2 and MMP-9 from untreated control rat renal myofibroblasts and cells treated with vehicle (10 μM NaOH) alone, RLX (16.8 nM) alone or increasing concentrations of DEA/NO (0.1–5 μM) alone, after 72 h in culture. Also shown are the mean ± SEM optical density (OD) of **(A)** pSmad2 and α-SMA (corrected for Smad2/3 loading); and **(B)** (latent and active) MMP-2 and MMP-9, as determined from densitometry measurements of the Western blots or zymographs. Numbers in parenthesis represent the number of independent experiments carried out in duplicate. *P < 0.05, **P < 0.01 vs. respective groups highlighted.

**FIGURE 2:**
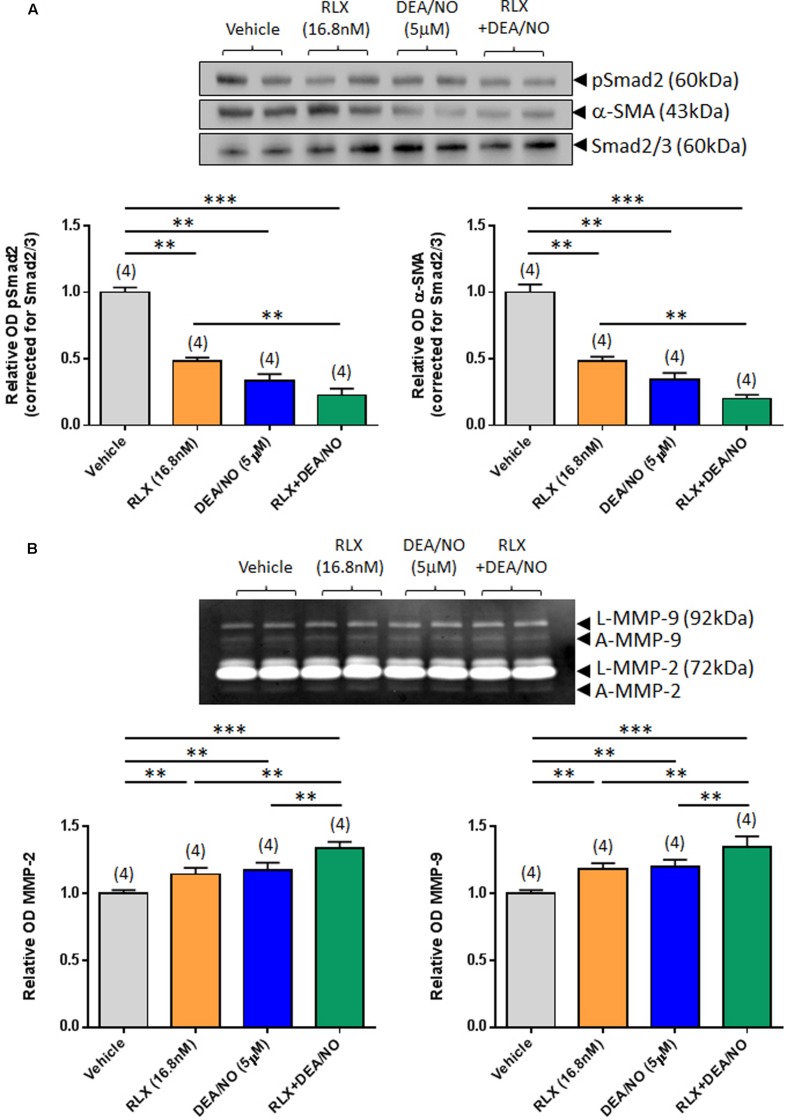
**The combined effects of RLX and DEA/NO on pSmad2, α-SMA, MMP-2 and MMP-9.** Shown are **(A)** representative (duplicate samples from one experiment) Western blots of pSmad2 and α-SMA; and **(B)** gelatin zymographs of latent (L) and active (A) MMP-2 and MMP-9 from vehicle (10 μM NaOH)-treated rat renal myofibroblasts and cells treated with RLX (16.8 nM) alone, DEA/NO (5 μM) alone or the combined effects of RLX (16.8 nM) alone and DEA/NO (5 μM) after 72 h in culture. Also shown are the mean ± SEM OD of **(A)** pSmad2 and α-SMA (corrected for Smad2/3 loading); and **(B)** (latent and active) MMP-2 and MMP-9, as determined from densitometry measurements of the Western blots or zymographs. Numbers in parenthesis represent the number of independent experiments carried out in duplicate. **P < 0.01, ***P < 0.001 vs. respective groups highlighted.

The effects of RLX (16.8 nM) on renal pSmad2, α-SMA expression, MMP-2 and MMP-9 levels were firstly compared to increasing concentrations of DEA/NO (0.1–5 μM) (**Figure 1**); and then combined with the highest concentration of DEA/NO evaluated (5 μM) (**Figure 2**). As previously demonstrated (Masterson et al., 2004; Mookerjee et al., 2009; Chow et al., 2012), RLX (16.8 nM) significantly inhibited pSmad2 and α-SMA expression by 35–50% (**Figures 1A** and **2A**), but increased both MMP-2 and MMP-9 levels by 25–30% (**Figures 1B** and **2B**) over 72 h in culture (all *P* < 0.05 vs. untreated control group). Although the magnitude of some of these RLX-induced effects were not as great as that reported previously (Masterson et al., 2004; Mookerjee et al., 2009; Chow et al., 2012), this may be due to the heterogeneity of primary cell cultures. In comparison, DEA/NO was found to concentration-dependently inhibit renal pSmad2 levels by ~10%, ~30% and ~33% when administered at 0.1, 1, and 5 μM, respectively, over 72 h in culture (all *P* < 0.05 vs. vehicle-treated group; **Figure 1A**). Likewise, DEA/NO significantly inhibited α-SMA expression by ~40 and ~60% at 1 and 5 μM, respectively, over the same time period (both *P* < 0.01 vs. untreated control and vehicle-treated groups; **Figure 1A**). On the other hand, the NO donor significantly increased MMP-2 and MMP-9 levels by ~33 and ~25%, respectively (both *P* < 0.05 vs. untreated control and vehicle-treated group), but only when administered at a concentration of 5 μM (**Figure 1B**).

Combining RLX (16.8 nM) with DEA/NO (5 μM) resulted in a further additive reduction of pSmad2 and α-SMA expression over 72 h in culture, by 50–55% over the effects of RLX alone (both *P* < 0.001 vs. vehicle-treated group; *P* < 0.01 vs. RLX alone-treated group; **Figure 2A**). Similarly, combining RLX and DEA/NO additively promoted both MMP-2 and MMP-9 levels over 72 h, by ~15% over the effects of RLX alone or DEA alone (both *P* < 0.001 vs. vehicle-treated group; *P* < 0.05 vs. RLX alone-treated group; *P* < 0.05 vs. DEA/NO alone-treated group; **Figure 2B**).

### The Effects of HXC and ODQ on the Anti-fibrotic Effects of DEA/NO and RLX

**FIGURE 3:**
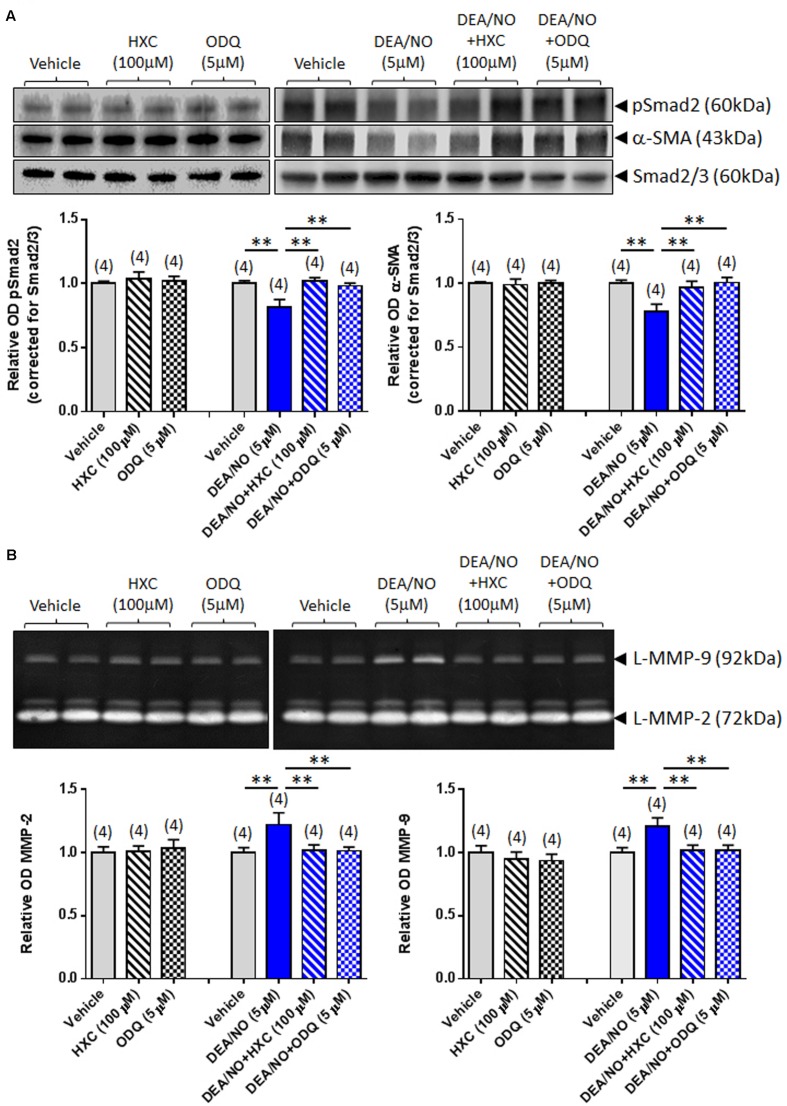
**The effects of HXC and ODQ on basal and DEA/NO-mediated changes of pSmad2, α-SMA, MMP-2 and MMP-9.** Shown are **(A)** representative (duplicate samples from one experiment) Western blots of pSmad2 and α-SMA; and **(B)** gelatin zymographs of latent (L) MMP-2 and MMP-9 from vehicle (10 μM NaOH)-treated rat renal myofibroblasts and cells treated with HXC (100 μM), ODQ (5 μM) alone, DEA/NO (5 μM) alone or the combined effects of DEA/NO (5 μM) and HXC (100 μM) or ODQ (5 μM), after 72 h in culture. Also shown are the mean ± SEM optical density (OD) of **(A)** pSmad2 and α-SMA (corrected for Smad2/3 loading); and **(B)** (latent) MMP-2 and MMP-9, as determined from densitometry measurements of the Western blots or zymographs. Numbers in parenthesis represent the number of independent experiments carried out in duplicate. **P < 0.01 vs. respective groups highlighted.

To further verify whether the effects of RLX were mediated via NO and sGC, its anti-fibrotic effects were next evaluated in the absence or presence of the NO scavenger, HXC (100 μM) and sGC inhibitor, ODQ (5 μM). The specificity of the effects of HXC and ODQ was first established in studies demonstrating that they were also able to completely abrogate the DEA/NO (5 μM)-induced down-regulation of pSmad2 and α-SMA expression (**Figure 3A**) in addition to the DEA/NO-mediated promotion of gelatinase expression (**Figure 3B**) (all *P* < 0.01 vs. DEA/NO alone-treated group), without affecting basal expression of these end-points over 72 h in culture.

**FIGURE 4:**
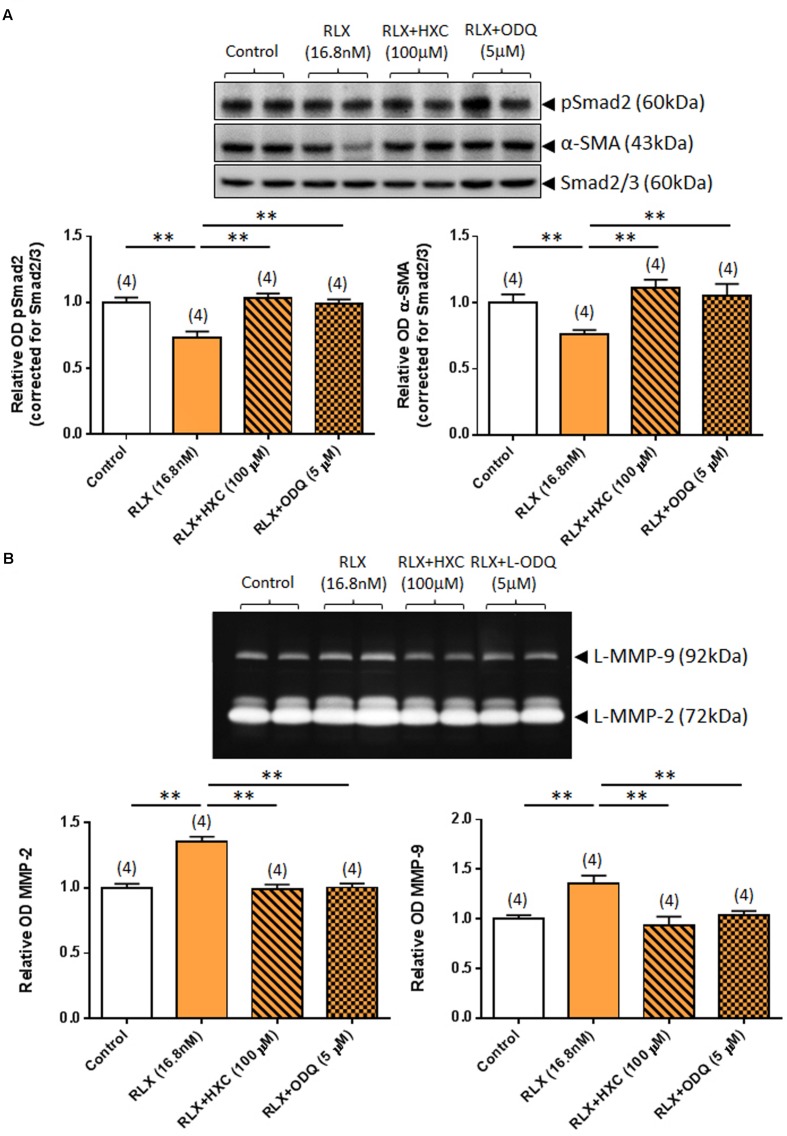
**The effects of HXC and ODQ on RLX-mediated changes of pSmad2, α-SMA, MMP-2 and MMP-9.** Shown are **(A)** representative (duplicate samples from one experiment) Western blots of pSmad2 and α-SMA; and **(B)** gelatin zymographs of latent (L) MMP-2 and MMP-9 from untreated (control) rat renal myofibroblasts and cells treated with RLX (16.8 nM) alone or the combined effects of RLX (16.8 nM) and HXC (100 μM) or ODQ (5 μM), after 72 h in culture. Also shown are the mean ± SEM optical density (OD) of **(A)** pSmad2 and α-SMA (corrected for Smad2/3 loading); and **(B)** (latent) MMP-2 and MMP-9, as determined from densitometry measurements of the Western blots or zymographs. Numbers in parenthesis represent the number of independent experiments carried out in duplicate. **P < 0.01 vs. respective groups highlighted.

Similarly, the ability of RLX (16.8 nM) to down-regulate pSmad2 and α-SMA expression was completely abrogated by either co-administration of the NO scavenger, HXC (100 μM) or the sGC inhibitor, ODQ (5 μM) (both *P* < 0.05 vs. RLX alone-treated group; **Figure 4A**) over 72 h. Likewise, the ability of RLX to promote MMP-2 and MMP-9 levels was completely abrogated by co-administration of HXC or ODQ over the same time period (both *P* < 0.01 vs. RLX alone-treated group; **Figure 4B**).

### The Effects of ODQ on the cGMP-Promoting Effects of RLX and DEA/NO

**FIGURE 5:**
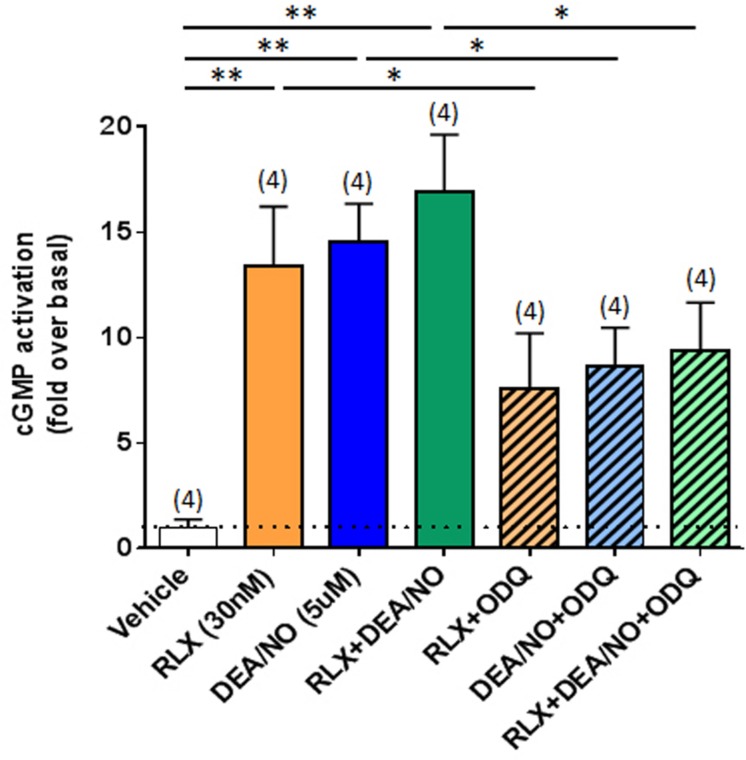
**The effects of ODQ on RLX ± DEA/NO-mediated changes in cGMP accumulation.** Shown are the mean ± SEM cGMP levels in rat renal myofibroblasts treated for 30 min with RLX (30 nM), DEA/NO (5 μM), or the combined effects of both; and sub-groups of correspondingly treated cells that were pre-exposed to ODQ (5 μM) for 15 min prior to administration of RLX + DEA/NO. Numbers in parenthesis represent the number of independent experiments carried out. *P < 0.05, **P < 0.01 vs. respective groups highlighted.

To further confirm that RLX was mediating its effects via cGMP, which is typically stimulated by sGC upon NO activation, direct measurement of cGMP accumulation from renal myofibroblasts was found to be strikingly increased by RLX (30 nM) or DEA/NO (5 μM) alone (by 12–13-fold; both *P* < 0.01 vs. basal (vehicle-treated) cGMP levels; **Figure 5**). Combining RLX and DEA/NO tended to increase cGMP levels further (by 16-fold), although this change was not statistically significant as compared to either treatment alone (**Figure 5**). These cGMP-promoting effects of RLX, DEA/NO or the combined effects of both were significantly inhibited by pre-administration of ODQ (5 μM) (all *P* < 0.05 vs. respective treatments alone; **Figure 5**).

## Discussion

This study aimed to further elucidate and confirm the molecular mechanisms by which the anti-fibrotic hormone, relaxin, inhibited the TGF-β1/pSmad2 axis (Heeg et al., 2005; Mookerjee et al., 2009). As the effects of RLX were enhanced in the presence of an NO donor, but abrogated in the presence of an NO scavenger, to the best of our knowledge, this study has demonstrated for the first time a direct contribution of NO to the TGF-β1-inhibitory effects of RLX. Furthermore, for the first time, we have shown that RLX directly stimulates cGMP upon its administration to rat renal myofibroblasts. Previous studies have shown that RLX up-regulates nNOS expression (of the NOS isoforms) in RXFP1-expressing rat renal myofibroblasts (Masterson et al., 2004; Mookerjee et al., 2009; Chow et al., 2012) and human cardiac fibroblasts (Sarwar et al., 2015). In this context, our findings have confirmed that relaxin signals through a RXFP1-pERK1/2-nNOS-NO-sGC-cGMP-dependent pathway to mediate its anti-fibrotic actions. Additionally, this study provided the first evidence that the NO donor, DEA/NO, could also induce anti-fibrotic actions via a sGC-cGMP-dependent pathway, leading to inhibition of TGF-β1 signal transduction (at the level of pSmad2) and subsequently, the pro-fibrotic influence of TGF-β1 on renal myofibroblast differentiation, while being able to promote collagen-degrading gelatinases (MMP-2 and MMP-9). Importantly, from a therapeutic perspective, our findings strongly suggest that combining RLX with NO donors would enhance its anti-fibrotic efficacy.

The TGF-β1-inhibitory effects of RLX have been well-documented in various (myo)fibroblast culture models *in vitro* (Unemori and Amento, 1990; Unemori et al., 1996; Samuel et al., 2004a; Heeg et al., 2005) and animal models of disease *in vivo* (Garber et al., 2001; Mookerjee et al., 2009; Samuel et al., 2011, 2014; Bennett et al., 2014). As detailed in Section “Introduction,” RLX binding and activation of its cognate receptor, RXFP1, on renal myofibroblasts results in the phosphorylation of ERK1/2 and activation of nNOS to inhibit pSmad2 (Mookerjee et al., 2009; Chow et al., 2012), in the absence of any direct effects on Smad3, Smad4, or Smad7 (Heeg et al., 2005), to disrupt TGF-β1 signal transduction. The findings from this study also support previous reports (Mookerjee et al., 2009; Chow et al., 2012) in demonstrating that the selective sGC inhibitor, ODQ (at 5 μM) was able to abolish the RLX-mediated inhibition of pSmad2 and α-SMA expression, in addition to its ability to promote gelatinase levels in the rat renal myofibroblasts studied. For the first time, they now demonstrate that the cGMP-promoting effects of RLX in these cells are also inhibited by ODQ. Based on previous studies in endothelial cells, it is likely that in renal myofibroblasts cGMP generated by stimulation of sGC suppresses Smad2/3 phosphorylation via a cGMP-dependent protein kinase 1 (PKG-1)-dependent manner (Saura et al., 2005). However, this is yet to be confirmed. Subsequently, inhibition of TGF-β1 releases various MMPs including MMP-2 and MMP-9 via an iNOS-dependent mechanism (Chow et al., 2012), which collectively contributes to the anti-fibrotic actions of RLX.

The therapeutic potential of targeting the NO-sGC-cGMP signaling pathway in kidney fibrosis was further highlighted by our findings with the NO donor, DEA/NO. DEA/NO, which spontaneously decomposes to generate NO with a half-life of ~2.5 min, exhibited similar anti-fibrotic actions to that of RLX (but at relatively higher concentration), which were sensitive to HXC and ODQ and thus, mediated via the NO-sGC-cGMP signaling pathway. These findings are supported by several *in vivo* and *in vitro* studies which have indicated that other NO donors can reduce fibrosis in multiple organs. For example, the NO donors molsidomine and sodium nitroprusside (SNP) were found to protect against liver fibrosis in rodents (Ozturk et al., 2002; Ali et al., 2012), while SNP and diethylenetetra-amine NONOate (DETA/NO) were reported to modulate human lung fibroblast proliferation (Liu et al., 2001). In myofibroblast culture models, it has also been reported that exogenous NO, donated by *S*-nitroso-*N*-acetyl penicillamine (SNAP), could inhibit myofibroblast proliferation, differentiation and collagen production (Vernet et al., 2002). In accordance with the current findings, these studies attributed the anti-fibrotic actions of NO donors to inhibition of the TGF-β1/Smad2 axis (Saura et al., 2005), suggesting a potential common mode of action of NO in myofibroblasts.

In contrast, other studies have demonstrated that the exogenous NO donor, DETA/NO (125–500 μM; *t*_1/2_ ~20 h), promoted TIMP-1, TGF-β1 and collagen synthesis in fibrosis-related disorders such as keloid (hypertrophic scar) and pulmonary fibrosis (Hsu et al., 2007). Moreover, while others have reported MMP-inhibitory effects of DETA/NO (500 μM) in a vascular model (Gurjar et al., 1999), our study suggested that DEA/NO (5 μM; *t*_1/2_ ~2.5 min) was able to promote MMP-2 and MMP-9 levels in myofibroblasts (which lack eNOS Mookerjee et al., 2009). These different findings with respect to the impact of NO donors on measures of fibrosis may reflect the use of NO donors with varying half-lives and at different concentrations, modulation of distinct downstream targets of cGMP and the study of diverse disease etiologies. Although further work is required to investigate which NO donors mediate the most efficacious anti-fibrotic effects in various pathological scenarios, our study demonstrates that DEA/NO at least exerts its anti-fibrotic actions through a similar mode of action to that of RLX, and in fact additively potentiates the anti-fibrotic effects of RLX. Collectively our data highlights the therapeutic potential of targeting the NO-sGC-cGMP signalling pathway in the treatment of fibrosis. In view of this, it would of great interest to evaluate the combined anti-fibrotic effects of RLX with the new generation NO-independent sGC stimulators (BAY 41-2272; Beyer et al., 2015) and/or cGMP analogs (8-Br-cGMP; Rivero-Vilches et al., 2003), the former demonstrating excellent safety and tolerability in phase III trials in patients with pulmonary hypertension. In particular, greater synergy may be achieved between RLX and BAY 41-2272, which target canonical and non-cononical (Beyer et al., 2015) TGF-β1 signal transduction pathways, respectively.

Despite the positive implications of these findings, there were a number of limitations with this study that need to be addressed in future investigations to further validate and translate the therapeutic potential of the treatments that were investigated. Firstly, the end-points measured were all completed from a cell culture model; albeit from cells isolated from injured rat kidneys (which are more reflective of their *in vivo* counterparts). Having said that, the findings obtained were consistent with data obtained from TGF-β1-stimulated human renal fibroblast cell lines (Heeg et al., 2005) and RLX-treated mice *in vivo* (Chow et al., 2014). Secondly, only one NO donor and one NO scavenger were used. However, our findings that HXC and ODQ were able to negate the effects of both the NO donor, DEA/NO and RLX strongly suggests that the effects of RLX were mediated via a NO-sGC-dependent mechanism. Given that NO scavengers and sGC inhibitors cannot be administered to the intact animal, they cannot be used to delineate the contribution of the NO-sGC-cGMP signaling pathway to the anti-fibrotic actions of RLX *in vivo*. Finally, while NO and sGC levels were not directly determined, we were able to demonstrate that the RLX- and DEA/NO-induced increase in cGMP accumulation was blocked by ODQ, confirming that RLX was able to signal through a NO-sGC-cGMP-dependent mechanism to mediate its anti-fibrotic actions *in vitro*.

Despite these limitations, this study confirmed at least at the *in vitro* level, that RLX mediates its overall anti-fibrotic actions in primary renal myofibroblasts via a NO-sGC-cGMP-dependent mechanism. As RLX is currently being assessed in phase III clinical trials for its cardio-protective and vasodilatory properties (Teerlink et al., 2013) and NO donors have been evaluated for their ability to treat fibrosis (Ozturk et al., 2002; Vernet et al., 2002; Ali et al., 2012), there is tremendous potential for combining these therapies to achieve added organ protection and reduced mortality. Extending this combination approach to the *in vivo* level will be key to translating the findings demonstrated in the current study.

## Author Contributions

Participated in research design: BK-H, CS. Conducted experiments: CW, BK-H, MK, SYA. Contributed reagents or tools: BK-H, TH, CS. Performed data analysis: CW, BK-H, MK, SA, TH, CS. Wrote or contributed to writing of manuscript: CW, BK-H, MK, TH, CS.

## Conflict of Interest Statement

The authors declare that the research was conducted in the absence of any commercial or financial relationships that could be construed as a potential conflict of interest.
